# Exercise-Mediated Regulation of Ferroptosis in Diabetic Cardiomyopathy: Mechanisms and Therapeutic Perspectives

**DOI:** 10.3390/ijms27209283

**Published:** 2026-10-19

**Authors:** Wenyan Bo, Qingxiang Guo

**Affiliations:** Institute of Physical Education, Shanxi University, Taiyuan 030006, China

**Keywords:** exercise, diabetic cardiomyopathy, ferroptosis, iron metabolism

## Abstract

Exercise is an important non-pharmacological intervention for the prevention and management of diabetes and its cardiovascular complications; however, the underlying mechanisms by which exercise confers cardiometabolic protection remain incompletely understood. Diabetic cardiomyopathy (DCM) is a major cardiovascular complication of diabetes, characterized by progressive myocardial structural and functional abnormalities associated with oxidative stress, dysregulated lipid metabolism, chronic inflammation, and miochondrial dysfunction. Ferroptosis, a form of regulated cell death characterized by iron-dependent lipid peroxidation, has emerged as a potential pathological link between metabolic disturbances and myocardial injury in DCM. Accumulating evidence suggests that exercise may modulate ferroptosis through multiple interconnected mechanisms, including restoration of iron homeostasis, suppression of lipid peroxidation, enhancement of endogenous antioxidant defenses, and improvement of mitochondrial function and quality control, thereby contributing to myocardial protection. In this review, we systematically summarize the cardioprotective benefits of exercise in the context of DCM and critically discuss the potential associations between exercise characteristics, including exercise modality, intensity, duration and the regulation of ferroptosis. By integrating current evidence on the molecular mechanisms underlying exercise-mediated ferroptosis regulation, this review provides a mechanistic framework for understanding how exercise may counteract ferroptosis in DCM and highlights the potential of ferroptosis-informed, precision exercise strategies for the prevention and management of diabetic cardiomyopathy.

## 1. Introduction

Diabetes mellitus (DM) is one of the three major chronic diseases posing a substantial global threat to human health. In 2024, approximately 589 million adults aged 20–79 years were living with DM worldwide, accounting for 11.1% of the adult population in this age group, or approximately one in every nine adults. This number is projected to increase to approximately 853 million by 2050, with the global prevalence expected to reach 12.9% [1]. DM is a chronic metabolic disorder driven by a complex interplay of genetic susceptibility, environmental factors, and lifestyle-related determinants, with persistent hyperglycemia and dysregulated glucose and lipid metabolism representing its cardinal pathological features [2]. Importantly, diabetic cardiomyopathy (DCM) is one of the most severe cardiovascular complications of diabetes [3]. It is characterized by structural and functional abnormalities of the myocardium that develop in the absence of other primary cardiovascular diseases, such as coronary artery disease, hypertension, or valvular heart disease [4,5]. Chronic hyperglycemia and metabolic disturbances can induce persistent myocardial injury through multiple interrelated mechanisms, including oxidative stress, lipotoxicity, chronic inflammation, accumulation of advanced glycation end products (AGEs), and mitochondrial dysfunction, ultimately contributing to adverse cardiac remodeling, myocardial fibrosis, and progressive deterioration of cardiac function [6]. Clinically, DCM is characterized by progressive interstitial fibrosis, left ventricular concentric hypertrophy, and impaired ventricular relaxation. Epidemiological evidence indicates that approximately 35% of individuals with type 2 diabetes mellitus (T2DM) exhibit varying degrees of cardiac dysfunction, while cardiovascular events remain a leading cause of diabetes-related mortality [7]. Current clinical management of diabetes primarily focuses on glycemic control and the mitigation of conventional cardiovascular risk factors, whereas effective interventions specifically targeting the complex pathological mechanisms underlying DCM remain limited. Therefore, elucidating the molecular mechanisms driving DCM and identifying novel therapeutic targets for its prevention and treatment have emerged as critical scientific priorities in the field of diabetic cardiovascular complications.

Ferroptosis is an iron-dependent form of regulated cell death that is typically initiated by disruption of cellular iron homeostasis, leading to enhanced iron-catalyzed Fenton reactions and excessive accumulation of lipid peroxides. Morphologically, ferroptotic cells exhibit characteristic mitochondrial abnormalities, including mitochondrial shrinkage, reduced or disrupted cristae, and rupture of the outer mitochondrial membrane, ultimately resulting in cell death [8,9]. Increasing evidence indicates that ferroptosis is involved in the pathogenesis of various cardiovascular diseases, including cardiomyopathies [10,11], myocardial infarction [12] and heart failure [13]. Notably, ferroptosis has also emerged as an important pathological mechanism implicated in the development and progression of DCM [14,15]. Studies in diabetic mice have demonstrated decreased expression of the key anti-ferroptotic regulators glutathione peroxidase 4 (GPX4) and solute carrier family 7 member 11 (SLC7A11), together with a reduced GSH/GSSG ratio, suggesting impaired glutathione-dependent antioxidant defense that may promote myocardial ferroptosis progression [16,17,18]. Consistently, clinical myocardial samples from patients with DM complicated by heart failure have demonstrated multiple molecular alterations associated with ferroptosis, including increased HMOX1, DMT1, and LPCAT3 and decreased GPX4, GSR, FLC, FPN1, and ACOT1 expression. These changes are consistent with enhanced iron availability and PUFA-containing phospholipid remodeling, together with impaired antioxidant defense and lipid peroxide detoxification, thereby favoring ferroptotic susceptibility in the diabetic failing human heart. Reduced ATF4/NRF2 signaling and increased CYBB expression may further contribute to this ferroptosis-prone state [19]. Importantly, pharmacological or genetic inhibition of cardiomyocyte ferroptosis has been shown to improve cardiac contractile function in mouse models of T2DM [20]. Collectively, these findings support ferroptosis as a mechanistically relevant component of DCM and suggest that its regulation may represent a potential therapeutic target.

Despite the rapid expansion of research on ferroptosis in cardiovascular and metabolic diseases, the current literature has not sufficiently addressed how ferroptosis in DCM can be modulated through clinically relevant non-pharmacological interventions. Previous reviews have predominantly focused on the molecular mechanisms of ferroptosis, its contribution to cardiovascular or diabetic complications, or pharmacological strategies targeting ferroptosis. Notably, relatively limited attention has been given to how exercise, as a modifiable and clinically relevant physiological stimulus, may regulate ferroptosis in the context of DCM and how exercise prescription may influence these effects. Exercise training is widely recognized as an effective non-pharmacological intervention for the prevention and management of diabetes and its cardiovascular complications. Regular exercise improves insulin sensitivity, attenuates oxidative stress and chronic inflammation, and contributes to the preservation of cardiac structure and function [21,22,23,24]. Emerging evidence suggests that modulation of ferroptosis may contribute to the cardioprotective effects of exercise in DCM [25,26]. Exercise may influence key ferroptosis-related processes by restoring cellular iron homeostasis, enhancing endogenous antioxidant defenses, attenuating lipid peroxidation, and maintaining myocardial mitochondrial redox homeostasis [25]. However, the available evidence has largely been examined within broader disease contexts and has not been systematically integrated according to the specific biological characteristics of exercise that may determine its effects on ferroptosis in diabetic cardiac injury. In particular, the potential relationships between exercise modality, intensity, duration, training volume, and other exercise characteristics and distinct ferroptosis-related processes remain insufficiently characterized. Different exercise prescriptions can elicit distinct metabolic, redox, inflammatory, and mitochondrial responses, which may consequently produce differential effects on ferroptosis-related signaling and cellular susceptibility to ferroptotic injury.

Against this background, the present review provides a focused and mechanistic synthesis of emerging evidence linking exercise, ferroptosis, and DCM. We systematically summarize the major molecular events through which ferroptosis contributes to diabetic myocardial injury, with particular emphasis on iron homeostasis, lipid peroxidation, antioxidant defense, and mitochondrial dysfunction. Further, we critically evaluate how exercise modulates these ferroptosis-related processes and discuss the potential upstream signaling pathways and inter-organ mediators involved in these adaptations. Importantly, we examine the potential relationships between exercise modality, intensity, duration, and other training characteristics and the magnitude or direction of ferroptosis-related responses. By integrating current mechanistic evidence with the biological characteristics of exercise, this review aims to advance the understanding of how exercise regulates ferroptosis in DCM and to provide a conceptual framework for future investigation of ferroptosis-informed precision exercise strategies and integrated approaches to the prevention and management of DCM. To inform this narrative review, we searched PubMed, Embase, and Google Scholar for relevant publications published between 2000 and 2026. The search terms included “ferroptosis,” “diabetic cardiomyopathy,” “exercise,” “diabetes,” “iron homeostasis”, “iron metabolism” and “cardiovascular disease.” We considered original studies involving human participants and animal models relevant to DCM, ferroptosis, and cardiovascular disease.

## 2. Mechanisms Underlying Ferroptosis-Mediated Diabetic Cardiomyopathy

### 2.1. Iron Metabolism Dysregulation and Iron Overload

Iron is an essential trace element for normal cardiomyocyte function and is involved in heme biosynthesis, iron–sulfur cluster assembly, oxidative phosphorylation, and redox enzyme activity. In cardiomyocytes, iron is mainly present as transferrin-bound iron, ferritin-stored iron, and the labile iron pool (LIP), which consists predominantly of redox-active Fe^2+^. The LIP represents an important link between iron metabolism and oxidative stress. Under physiological conditions, iron uptake, intracellular transport, storage, utilization, and export are tightly coordinated to maintain iron homeostasis and meet mitochondrial metabolic demands while limiting iron-mediated oxidative damage [14]. Disruption of this balance can expand the LIP and increase redox-active Fe^2+^, thereby promoting reactive oxygen species (ROS) generation through Fenton chemistry and increasing susceptibility to lipid peroxidation and ferroptosis. The accumulation of lipid peroxides can progressively overwhelm cellular antioxidant defenses. When the capacity to detoxify lipid peroxidation products becomes insufficient, uncontrolled lipid peroxidation ensues, ultimately disrupting membrane integrity and promoting ferroptotic cell death [27,28]. This remodeling of intracellular iron homeostasis may increase cardiomyocyte susceptibility to ferroptosis and represents an important upstream link between diabetic metabolic stress and myocardial injury.

Iron uptake by cardiomyocytes is mainly mediated by the transferrin (TF)/transferrin receptor 1 (TFR1) pathway. Circulating Fe^3+^ binds to TF and is internalized through TFR1-mediated endocytosis. Within endosomes, STEAP family metalloreductases reduce Fe^3+^ to Fe^2+^, which is then released into the cytosolic LIP through divalent metal transporter 1 (DMT1) [29]. Other transporters, including DMT1, ZIP8/ZIP14, and L-type and T-type calcium channels, may also contribute to non-transferrin-bound iron uptake [30,31]. Under diabetic and hyperglycemic conditions, oxidative stress, inflammation, and hypoxic stress may disrupt these pathways and promote LIP expansion. However, evidence for myocardial iron loading in DCM remains inconsistent. Some diabetic models show increased Fe^2+^ or LIP levels [32], whereas others show no significant change in total myocardial iron content [33]. Thus, expansion of the LIP and remodeling of intracellular iron homeostasis may be more relevant to ferroptosis in DCM than total myocardial iron accumulation.

Impaired iron export also contributes to cardiac iron dysregulation. Ferroportin 1 (FPN1) is the major cellular iron exporter and transports cytosolic Fe^2+^ across the plasma membrane. Extracellular Fe^2+^ can then be oxidized to Fe^3+^ by ferroxidases such as ceruloplasmin and re-associated with TF [34,35]. Hepcidin promotes FPN1 internalization and lysosomal degradation, thereby restricting iron export and increasing cellular iron retention. Under chronic inflammation and metabolic stress associated with diabetes, dysregulation of the hepcidin–FPN1 axis may impair myocardial iron efflux and further expand the LIP. Enhanced iron uptake together with impaired iron export may therefore contribute to the accumulation of redox-active iron in DCM [36]. Ferritinophagy-mediated iron mobilization provides another important link between autophagy and ferroptosis. Intracellular iron is mainly stored in ferritin, which consists of ferritin heavy chain 1 (FTH1) and ferritin light chain (FTL), thereby limiting the redox activity of free iron. Nuclear receptor coactivator 4 (NCOA4) selectively recognizes FTH1 and delivers ferritin to autophagosomes and lysosomes for degradation, releasing stored iron and expanding the LIP [37]. Excessive NCOA4-mediated ferritinophagy may increase intracellular iron availability, promote ROS generation and lipid peroxidation, and enhance ferroptotic susceptibility [38]. In diabetic myocardial ischemia/reperfusion injury, inhibition of DNMT1 has been reported to attenuate ferroptosis by regulating NCOA4-mediated ferritinophagy [39]. Recent evidence also indicates that DCM is associated with increased myocardial fibrosis, ferroptosis, ROS production, and lipid accumulation, together with enhanced NCOA4-associated ferritinophagy and impaired Parkin-dependent mitophagy. Inhibition of NCOA4-mediated ferritinophagy may improve cardiac remodeling through regulation of the MITOL/March5–Parkin pathway [40]. These findings suggest that ferritinophagy and mitochondrial quality control may interact to regulate ferroptotic susceptibility in DCM.

### 2.2. Redox Imbalance and Impaired Antioxidant Defense

Ferroptosis is determined not only by intracellular iron accumulation and the availability of oxidation-prone lipid substrates but also by the capacity of cells to maintain redox homeostasis and detoxify lipid peroxides. Among the endogenous antioxidant systems, the system Xc^−^–glutathione (GSH)–glutathione peroxidase 4 (GPX4) axis is a central defense mechanism against lipid peroxidation and ferroptosis. System Xc^−^, composed of solute carrier family 7 member 11 (SLC7A11) and solute carrier family 3 member 2 (SLC3A2), imports extracellular cystine in exchange for glutamate, thereby providing cysteine for GSH synthesis. Intracellular cysteine can also be supplied through the transsulfuration pathway or alternative transporters such as SLC1A11 [41,42]. GSH serves both as a major intracellular antioxidant and as the essential reducing cofactor for GPX4, which reduces membrane phospholipid hydroperoxides (L-OOH) to relatively stable lipid alcohols (L-OH), thereby limiting the propagation of lipid peroxidation [28]. GPX4 activity depends on its catalytic selenocysteine, making selenium availability important for maintaining its structure and function [43,44]. When cystine uptake, GSH synthesis, or GPX4 activity is impaired, the detoxification of lipid peroxides is compromised, leading to their progressive accumulation and increased ferroptotic susceptibility [45]. This defense system is particularly important in DCM, where chronic hyperglycemia, lipotoxicity, inflammation, and oxidative stress place sustained pressure on cellular antioxidant capacity. In diabetic mouse models, dysfunction of the SLC7A11/GSH/GPX4 axis has been associated with mitochondrial iron overload and increased ROS accumulation [46], whereas modulation of this pathway to suppress ferroptosis can improve cardiac function [47]. Collectively, these findings indicate that the system Xc^−^–GSH–GPX4 axis is a critical determinant of lipid redox homeostasis and ferroptotic susceptibility in DCM.

Nuclear factor erythroid 2-related factor 2 (NRF2) is a master regulator of cellular redox homeostasis and a central component of the endogenous antioxidant defense system. Under basal conditions, NRF2 is continuously targeted for ubiquitination and proteasomal degradation through its interaction with Kelch-like ECH-associated protein 1 (Keap1). Under oxidative or electrophilic stress, this degradation is inhibited, allowing NRF2 to accumulate and translocate into the nucleus, where it binds antioxidant response elements (AREs) and induces the expression of cytoprotective genes, including heme oxygenase-1 (HO-1), enzymes involved in glutathione (GSH) metabolism, and antioxidant enzymes such as superoxide dismutase (SOD) [48]. In DCM, persistent hyperglycemia, lipotoxicity, and mitochondrial dysfunction can increase oxidative stress and challenge NRF2-dependent antioxidant defenses. Experimental evidence indicates that NRF2 deficiency exacerbates diabetes-associated myocardial oxidative injury, whereas activation of NRF2 signaling can attenuate oxidative stress, inflammation, fibrosis, and adverse myocardial remodeling [7,17,49,50].

NRF2 also represents an important regulatory link between oxidative stress and ferroptosis. It can promote the expression of iron-storage proteins such as ferritin heavy chain 1 (FTH1), thereby limiting the expansion of the LIP, while enhancing SLC7A11-mediated cystine uptake, GSH biosynthesis, and GPX4-dependent detoxification of lipid peroxides [51]. Through additional effects on lipid metabolism and mitochondrial homeostasis, NRF2 may further restrict ROS generation and lipid peroxidation [51,52]. However, the protective role of NRF2 may depend on disease stage and cellular context. Transient NRF2 activation during early metabolic stress may provide adaptive protection, whereas persistent hyperglycemia, lipotoxicity, and inflammation may overwhelm this response, leading to impaired antioxidant defense, disrupted iron homeostasis, and increased susceptibility to ferroptosis [53]. Consistent with this concept, pharmacological activation of NRF2 has been reported to reduce myocardial oxidative injury and fibrosis and improve cardiac function [54,55]. Moreover, curcumin-induced NRF2 nuclear translocation increased HO-1 expression, attenuated GPX4 depletion, and reduced high glucose-induced ferroptosis in cardiomyocytes [56]. Crosstalk between NRF2, autophagy, and lipid metabolism may further influence DCM progression [57].

Overall, NRF2 functions as a key regulatory hub connecting redox balance, iron homeostasis, lipid peroxidation, and mitochondrial function. Although preclinical evidence supports NRF2 activation as a potential strategy for limiting ferroptosis and myocardial injury in DCM, its therapeutic efficacy and translational relevance in patients remain to be established because direct evidence from human studies is still limited.

### 2.3. Lipid Metabolic Remodeling and Lipid Peroxidation

Dysregulated lipid metabolism is a major pathological feature of DCM. Lipotoxicity refers to the excessive accumulation of free fatty acids (FFAs) and their bioactive metabolites in non-adipose tissues, leading to metabolic disturbances and cellular dysfunction [58]. Under chronic hyperglycemia and insulin resistance, myocardial fatty acid uptake is increased, whereas fatty acid oxidation and mitochondrial metabolic capacity may be impaired, resulting in sustained lipid accumulation in cardiomyocytes [59]. However, lipid accumulation alone does not necessarily induce ferroptosis. Ferroptotic susceptibility depends largely on the availability of oxidation-prone membrane lipids and the capacity to detoxify the resulting lipid peroxides. In this context, fatty acid composition and membrane phospholipid remodeling are critical determinants. Acyl-CoA synthetase long-chain family member 4 (ACSL4) activates polyunsaturated fatty acids (PUFAs), including arachidonic acid (AA) and adrenic acid (AdA), to form PUFA-CoA derivatives, which are subsequently incorporated into membrane phospholipids, particularly phosphatidylethanolamine (PE), through lysophosphatidylcholine acyltransferase 3 (LPCAT3) [60,61]. Because PUFAs contain multiple double bonds and bis-allylic hydrogen atoms that are highly susceptible to oxidation, their enrichment in membrane phospholipids increases lipid peroxidation and ferroptotic susceptibility. Thus, ACSL4–LPCAT3-mediated phospholipid remodeling provides an important metabolic link between lipid dysregulation and ferroptosis [62].

Lipid peroxidation is a central execution process of ferroptosis. In the presence of iron and ROS, PUFA-containing membrane phospholipids undergo enzymatic oxidation mediated by lipoxygenases (LOXs) and non-enzymatic autoxidation, generating lipid radicals, lipid peroxyl radicals, and lipid hydroperoxides (L-OOH), which can further produce reactive aldehydes such as malondialdehyde (MDA) and 4-hydroxynonenal (4-HNE) [63]. Excessive accumulation of oxidized PUFA-containing phospholipids, particularly oxidized PE species, disrupts membrane lipid homeostasis and promotes irreversible cellular injury [64]. Under physiological conditions, glutathione peroxidase 4 (GPX4) reduces L-OOH to relatively stable lipid alcohols, thereby limiting lipid radical propagation [65]. In DCM, persistent hyperglycemia, lipid overload, mitochondrial dysfunction, and oxidative stress can increase the burden of lipid peroxidation, while impairment of the SLC7A11–glutathione (GSH)–GPX4 system limits lipid peroxide detoxification [66,67,68]. Reactive aldehydes such as 4-HNE may further modify proteins involved in antioxidant defense and mitochondrial function, amplifying oxidative damage [69]. Oxidation of phosphatidylcholine species containing two PUFA acyl chains, particularly those enriched in mitochondria, may also contribute to ferroptotic signaling [70]. Overall, in the pathological environment of DCM, chronic hyperglycemia and lipid overload promote the accumulation and remodeling of oxidation-prone lipids, while mitochondrial and redox dysfunction increase ROS generation. Concurrently, dysregulated iron metabolism provides redox-active catalytic conditions, and impaired antioxidant defenses limit lipid peroxide detoxification. The convergence of these processes promotes sustained lipid peroxidation and ultimately contributes to ferroptotic cardiomyocyte injury.

### 2.4. Mitochondrial Dysfunction

Mitochondria are central organelles responsible for energy metabolism, fatty acid oxidation, and redox homeostasis in cardiomyocytes and represent an important regulatory hub linking mitochondrial dysfunction to ferroptosis. Given the high energy demands of the myocardium, cardiomyocyte ATP production depends largely on mitochondrial oxidative phosphorylation and fatty acid oxidation. Mitochondrial dysfunction can therefore profoundly disrupt metabolic and redox homeostasis. Increasing evidence suggests that mitochondria are not only an important source of ferroptosis-associated ROS [71], but also regulate ferroptotic susceptibility through their effects on iron homeostasis, lipid metabolism, oxidative phosphorylation, and cellular redox status [72]. Conversely, preservation of mitochondrial biogenesis, membrane integrity, and oxidative phosphorylation may limit ROS accumulation and lipid peroxidation, thereby reducing ferroptotic stress.

In DCM, chronic hyperglycemia, insulin resistance, and lipid metabolic disturbances impose sustained stress on mitochondria, leading to electron transport chain dysfunction, loss of mitochondrial membrane potential, impaired ATP production, and excessive ROS generation [73]. Impaired mitochondrial fatty acid oxidation may further promote lipid intermediate accumulation and exacerbate oxidative stress and lipid peroxidation [74,75]. Thus, mitochondrial dysfunction and ferroptosis may form a bidirectional amplification loop rather than a simple unidirectional relationship. Damaged mitochondria can increase ROS production and lipid peroxidation, whereas persistent lipid peroxidation can impair mitochondrial membrane integrity and oxidative phosphorylation, further aggravating oxidative damage and ferroptotic progression [76]. Consistent with this concept, LGR6 promotes mitochondrial biogenesis through the STAT3–PGC-1α pathway, and LGR6 deficiency exacerbates mitochondrial dysfunction and myocardial ferroptosis in diabetic mice, whereas cardiac-specific LGR6 overexpression attenuates these abnormalities. Inhibition of STAT3 or activation of PGC-1α further supports the involvement of the LGR6–STAT3–PGC-1α axis in maintaining mitochondrial homeostasis and limiting ferroptotic injury [77].

Mitochondrial iron homeostasis provides another important link between mitochondrial dysfunction and ferroptosis. Excessive mitochondrial iron accumulation can expand the mitochondrial labile iron pool, promote ROS generation, and enhance lipid peroxidation. Consistent with this mechanism, myocardial iron overload has been associated with mitochondrial iron accumulation, oxidative stress, mitochondrial dysfunction, and increased cardiomyocyte ferroptosis. Parkin has been reported to alleviate iron overload-induced ferroptosis by promoting ACSL4 ubiquitination, thereby reducing mitochondrial dysfunction and lipid peroxidation. p53 may transcriptionally repress Parkin expression, suggesting a potential p53–Parkin–ACSL4 regulatory axis in ferroptotic injury [78]. However, the specific contribution of this pathway to DCM remains to be established.

Mitochondrial DNA (mtDNA) may also contribute to ferroptosis regulation. Although nuclear-encoded factors have been extensively studied, impaired mtDNA synthesis and mtDNA damage can increase cellular susceptibility to ferroptosis [79,80,81]. Notably, mitochondrial α/β-hydrolase domain-containing protein 11 (ABHD11)-mediated mtDNA transcription has been reported to facilitate mitochondria–nucleus communication, reduce lipid peroxidation and ferroptosis in cardiomyocytes, and promote cardiac recovery after myocardial infarction [82]. These findings suggest that mtDNA integrity and transcriptional activity may represent an additional layer of mitochondrial regulation of ferroptotic susceptibility.

### 2.5. Ferroptosis, Inflammation, Endoplasmic Reticulum Stress, and Myocardial Fibrosis

DCM is not solely a consequence of metabolic abnormalities within cardiomyocytes but involves a complex interplay among chronic inflammation, endoplasmic reticulum stress (ERS), regulated cell death, and extracellular matrix (ECM) remodeling. Increasing evidence indicates that ferroptosis is closely interconnected with these pathological processes through bidirectional regulatory mechanisms [83]. In particular, under persistent hyperglycemic, lipotoxic, and oxidative stress conditions, lipid peroxidation products and damage-associated signals generated during ferroptotic injury may further activate inflammatory and stress responses, whereas chronic inflammation and ERS can exacerbate iron dysregulation and lipid peroxidation, thereby establishing a self-reinforcing pathological network that contributes to DCM progression. 

Chronic low-grade inflammation is a well-established feature of DCM. Persistent hyperglycemia, lipotoxicity, and oxidative stress activate inflammatory pathways, including nuclear factor-κB (NF-κB) and the NOD-like receptor family pyrin domain-containing 3 (NLRP3) inflammasome, leading to increased production of tumor necrosis factor-α (TNF-α), interleukin (IL)-1β, and IL-6 and contributing to myocardial injury and dysfunction [84]. Experimental studies in diabetic cardiac models have also linked ferroptosis with inflammatory activation, although the direction and molecular basis of this interaction remain incompletely defined. Mechanistically, inflammatory signaling may impair system Xc^−^ activity and glutathione (GSH) availability, thereby weakening GPX4-dependent lipid peroxide detoxification and increasing ferroptotic susceptibility [85]. Conversely, oxidative stress and lipid peroxidation associated with ferroptotic injury may provide damage-associated signals that activate inflammatory pathways. For example, mitochondrial ROS, oxidized mitochondrial DNA, and damage-associated molecular patterns (DAMPs) can activate NLRP3 or NF-κB signaling in experimental systems [86,87]. However, evidence that specific cytokines such as IL-1β or TNF-α directly regulate ferroptosis-associated molecules, including ACSL4 or transferrin receptor 1 (TFR1), is derived largely from non-DCM models [88,89]. Thus, the reciprocal relationship between inflammation and ferroptosis is biologically plausible and supported by experimental evidence, but its precise contribution to DCM requires further validation in disease-specific models.

The endoplasmic reticulum (ER) is highly sensitive to metabolic disturbances caused by hyperglycemia and lipid overload. Sustained accumulation of misfolded proteins activates the unfolded protein response (UPR), involving the PERK–eIF2α–ATF4, IRE1α–XBP1, and ATF6 pathways [90,91]. ERS is well established as a contributor to cardiomyocyte dysfunction and myocardial remodeling in diabetes. Evidence from DCM models also indicates that ERS is associated with oxidative stress, inflammation, and cardiomyocyte injury. Ferroptosis may intersect with ERS through changes in cellular redox balance, lipid metabolism, and antioxidant capacity. In experimental models outside DCM, persistent ERS has been shown to increase ROS and lipid peroxidation and thereby enhance ferroptotic susceptibility, whereas ferroptotic lipid peroxidation and mitochondrial dysfunction can further disrupt ER homeostasis [32,92,93]. These observations provide a mechanistic basis for potential crosstalk between ERS and ferroptosis in DCM. Nevertheless, direct evidence demonstrating a causal ERS–ferroptosis relationship specifically in DCM remains limited, and further studies are needed to determine whether this interaction is a primary driver or a secondary consequence of diabetic myocardial injury.

Myocardial fibrosis is a major pathological feature of DCM and results from excessive ECM deposition and collagen accumulation. Hyperglycemia, oxidative stress, and inflammation promote cardiac fibroblast activation and collagen synthesis through profibrotic pathways, particularly transforming growth factor-β (TGF-β)/Smad signaling [94]. Direct evidence from diabetic cardiac models suggests that ferroptosis is associated with myocardial fibrosis. For example, studies of diabetic cardiomyopathy have reported increased lipid peroxidation and ferroptosis-related alterations together with collagen accumulation, while pharmacological or genetic enhancement of antioxidant/ferroptosis-regulatory pathways can attenuate myocardial injury and fibrosis [95].

However, the molecular mechanisms linking ferroptosis to fibroblast activation are less well established in DCM. Evidence from other fibrotic disease models suggests that lipid peroxidation and ROS may enhance TGF-β/Smad signaling and promote fibroblast activation, whereas TGF-β signaling may impair the system Xc^−^–GSH–GPX4 axis and increase cellular susceptibility to ferroptosis [96,97,98]. These findings support a potential bidirectional relationship, but the extent to which ferroptosis directly drives cardiac fibroblast activation or ECM deposition in DCM remains to be determined.

Importantly, cellular senescence is increasingly recognized as an important feature of DCM, characterized by persistent cell-cycle arrest, mitochondrial dysfunction, and a senescence-associated secretory phenotype (SASP) that may contribute to chronic inflammation and adverse myocardial remodeling [99]. Ferroptosis and senescence share several pathological triggers, including oxidative stress, mitochondrial dysfunction, iron dysregulation, and impaired antioxidant capacity [100,101]. Recent studies suggest that senescent cells may be particularly vulnerable to ferroptosis because of increased oxidative stress, altered iron metabolism, and greater dependence on GPX4-mediated lipid peroxide detoxification [102]. However, most evidence linking ferroptosis and senescence has been obtained from non-DCM models, and direct evidence establishing a causal relationship between these processes in diabetic hearts remains limited. Thus, ferroptosis and senescence may represent interconnected but distinct stress responses that converge on oxidative injury, inflammation, and myocardial remodeling.

**Figure 1:**
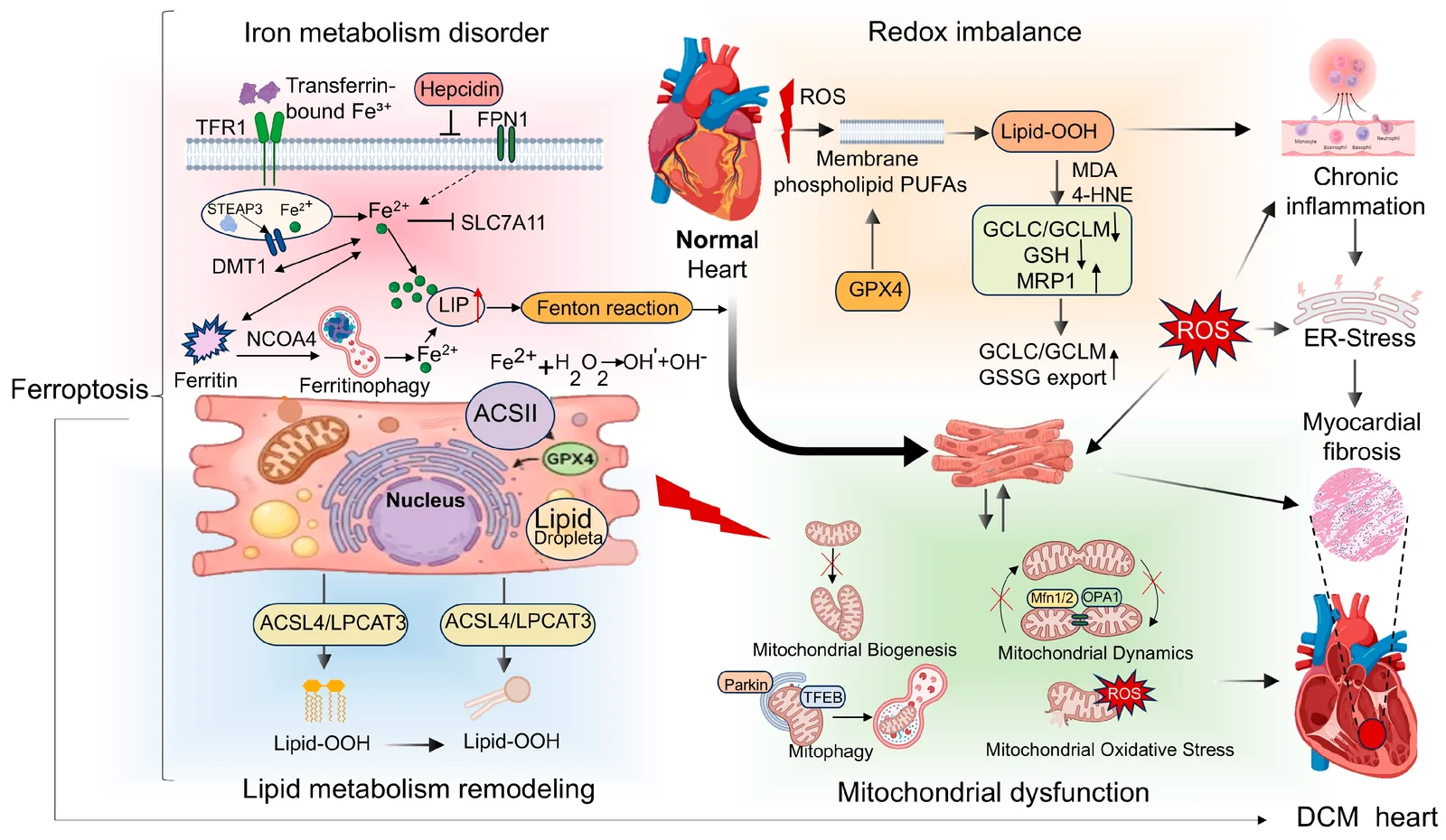
Ferroptosis and its interconnected mechanisms in the progression of diabetic cardiomyopathy. Iron accumulation driven by increased iron uptake, impaired FPN1-mediated iron export, and NCOA4-mediated ferritinophagy expands the labile iron pool, promoting ROS generation and lipid peroxidation. These changes, together with redox imbalance and mitochondrial dysfunction, contribute to chronic inflammation, myocardial fibrosis, and DCM progression. Vertical arrows denote the direction of change, with upward arrows (↑) indicating upregulation or enhancement and downward arrows (↓) indicating downregulation or inhibition. Abbreviations: ACSL4—acyl-CoA synthetase long-chain family member 4; DCM—diabetic cardiomyopathy; DMT1—divalent metal transporter 1; ER—endoplasmic reticulum; FPN1—ferroportin 1; GCLC—glutamate-cysteine ligase catalytic subunit; GCLM—glutamate-cysteine ligase modifier subunit; GPX4—glutathione peroxidase 4; GSH—glutathione; GSSG—glutathione disulfide; LIP—labile iron pool; LPCAT3—lysophosphatidylcholine acyltransferase 3; MDA—malondialdehyde; MFN2—mitofusin 2; MRP1—multidrug resistance-associated protein 1; NCOA4—nuclear receptor coactivator 4; OPA1—optic atrophy 1; PUFA—polyunsaturated fatty acid; ROS—reactive oxygen species; SLC7A11—solute carrier family 7 member 11; STEAP3—six-transmembrane epithelial antigen of the prostate 3; TFEB—transcription factor EB; TFR1—transferrin receptor 1; 4-HNE—4-hydroxynonenal.

Overall, ferroptosis in DCM is driven by the convergence of iron dysregulation, lipid metabolic remodeling, oxidative stress, and impaired antioxidant defenses. Increased redox-active iron provides the catalytic conditions for ROS generation, while altered lipid metabolism promotes the accumulation of PUFA-containing phospholipids that are susceptible to peroxidation. Mitochondrial dysfunction further exacerbates ROS production, lipid metabolic disturbances, and redox imbalance, whereas impairment of the SLC7A11–GSH–GPX4 and NRF2-dependent defense systems limits the detoxification of lipid peroxides. In parallel, chronic inflammation, endoplasmic reticulum stress, myocardial fibrosis, and cellular senescence may interact with ferroptosis and contribute to the progression of diabetic myocardial injury. These interconnected processes form a self-reinforcing network that increases ferroptotic susceptibility and promotes cardiomyocyte injury and adverse cardiac remodeling in DCM. Therefore, targeting the coordinated regulation of iron homeostasis, lipid metabolism, mitochondrial function, and antioxidant defense may represent an important strategy for limiting ferroptosis and mitigating DCM progression (Figure 1).

## 3. Mechanisms Underlying Exercise-Mediated Regulation of Ferroptosis

**Figure 2:**
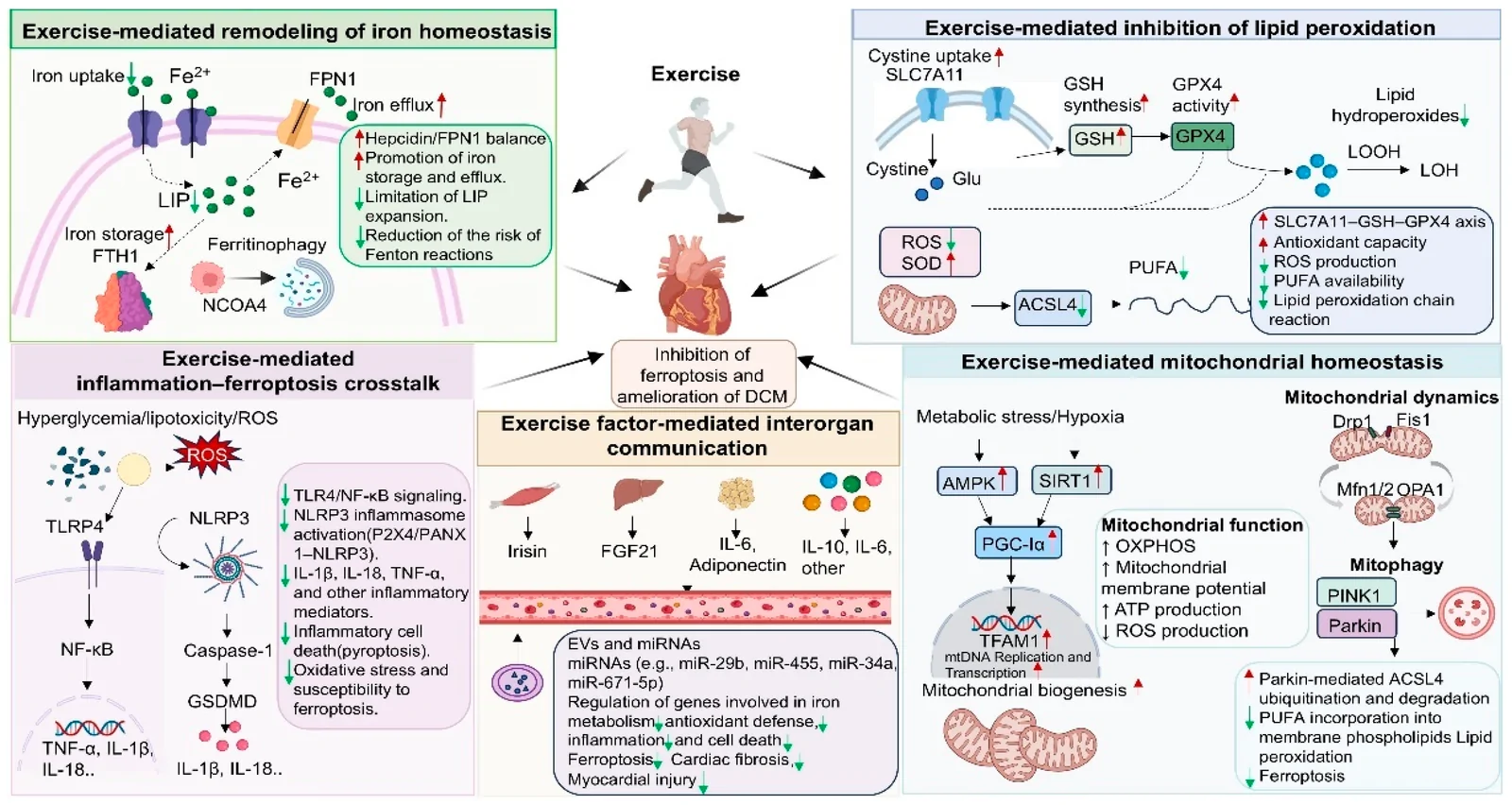
Exercise-mediated regulation of ferroptosis in diabetic cardiomyopathy Exercise may suppress ferroptosis and ameliorate DCM through coordinated regulation of myocardial iron homeostasis, lipid peroxidation, inflammation, and mitochondrial quality control. Exercise promotes iron efflux and storage while limiting labile iron accumulation, enhances the SLC7A11–GSH–GPX4 antioxidant system, reduces PUFA-dependent lipid peroxidation, and attenuates inflammation–ferroptosis crosstalk. Meanwhile, exercise improves mitochondrial biogenesis, dynamics, and mitophagy, while exercise-induced myokines, extracellular vesicles, and microRNAs may facilitate interorgan communication and further regulate ferroptosis-related pathways. Collectively, these effects establish a multifaceted protective network that limits ferroptotic injury and contributes to the prevention of myocardial fibrosis and adverse cardiac remodeling. Vertical arrows denote the direction of change, with upward arrows (↑) indicating upregulation or enhancement and downward arrows (↓) indicating downregulation or inhibition. Abbreviations: ACSL4—acyl-CoA synthetase long-chain family member 4; AMPK—AMP-activated protein kinase; DCM—diabetic cardiomyopathy; Drp1—dynamin-related protein 1; EVs—extracellular vesicles; FGF21—fibroblast growth factor 21; Fis1—fission 1 protein; FPN1—ferroportin 1; FTH1—ferritin heavy chain 1; GPX4—glutathione peroxidase 4; GSDMD—gasdermin D; GSH—glutathione; IL-6—interleukin-6; IL-10—interleukin-10; IL-18—interleukin-18; IL-1β—interleukin-1 beta; LIP—labile iron pool; LOOH—lipid hydroperoxides; LOH—lipid alcohols; Mfn1/2—mitofusin 1/2; miRNAs—microRNAs; NCOA4—nuclear receptor coactivator 4; NF-κB—nuclear factor kappa B; NLRP3—NOD-like receptor family pyrin domain-containing 3; OPA1—optic atrophy 1; OXPHOS—oxidative phosphorylation; P2X4—purinergic receptor P2X 4; PANX1—pannexin 1; PGC-1α—peroxisome proliferator-activated receptor gamma coactivator 1-alpha; PINK1—PTEN-induced putative kinase 1; PUFA—polyunsaturated fatty acid; ROS—reactive oxygen species; SLC7A11—solute carrier family 7 member 11; SOD—superoxide dismutase; SIRT1—sirtuin 1; TLR4—Toll-like receptor 4; TNF-α—tumor necrosis factor-alpha.

The cardioprotective effects of exercise in DCM may involve multiple molecular and cellular processes rather than a single signaling pathway, potentially reshaping the myocardial microenvironment associated with ferroptosis susceptibility. Evidence from DCM models and other experimental systems suggests that exercise may influence myocardial iron homeostasis, the SLC7A11–GSH–GPX4 antioxidant defense system, inflammatory responses, lipid peroxidation, and mitochondrial function and quality control (Table 1). However, the extent to which these mechanisms contribute directly to ferroptosis regulation in DCM remains to be established. In addition, exercise-induced myokines, extracellular vesicles, and non-coding RNAs may mediate interorgan communication among skeletal muscle, the liver, adipose tissue, and the heart. Although these findings suggest potential systemic mechanisms linking exercise to myocardial ferroptosis regulation, their specific roles in DCM require further direct validation (Figure 2).

### 3.1. Exercise-Mediated Remodeling of Myocardial Iron Homeostasis and Lipid Redox Balance

Disruption of iron homeostasis and impaired lipid peroxide detoxification are key processes involved in ferroptosis in DCM. Aerobic exercise may counteract these processes by regulating myocardial iron handling and strengthening antioxidant defenses. In DCM models, aerobic exercise improved cardiac function, increased the expression of GPX4, FTH1, SLC7A11, and FSP1, and decreased the expression of ACSL4 and prostaglandin-PTGS2, together with myocardial Fe^2+^ levels [25,26]. These findings suggest that exercise may alleviate ferroptosis-related changes in DCM by limiting iron availability and enhancing cellular defenses against lipid peroxidation. Nevertheless, changes in ferroptosis-related markers alone do not establish that ferroptosis suppression directly mediates the cardioprotective effects of exercise. Ferritinophagy may represent an additional mechanism linking iron homeostasis to ferroptosis in DCM. NCOA4-mediated ferritin degradation can increase intracellular iron availability, whereas NCOA4 inhibition alleviates high-glucose-induced ferroptosis in cardiomyocytes [37]. However, whether exercise regulates NCOA4-mediated ferritinophagy in DCM remains unknown. Furthermore, although increased myocardial Fe^2+^ levels have been reported in DCM models, direct evidence demonstrating an expansion of the LIP remains lacking. Intracellular Fe^2+^ levels and the LIP are related but distinct measures, as not all intracellular Fe^2+^ belongs to the readily chelatable, redox-active pool. Therefore, increased Fe^2+^ levels suggest altered iron homeostasis but do not establish that the LIP is expanded. Direct LIP measurements are needed to determine whether changes in redox-active iron availability contribute to ferroptosis in DCM.

Studies of metabolic and other cardiac injuries provide further evidence that exercise can regulate myocardial iron homeostasis. Long-term aerobic exercise reduced myocardial iron accumulation, increased the expression of FPN1, FTH1, GPX4, and SLC7A11, decreased ACSL4 expression, and improved cardiac function [103,108]. Kordi et al. [109] reported that regular exercise restored the hepcidin–FPN1 balance and promoted iron release from macrophages and cardiomyocytes. These findings suggest that exercise may influence iron export and storage, thereby limiting the LIP. However, total myocardial iron content does not necessarily reflect changes in the LIP or mitochondrial iron pools, and these regulatory effects require validation in DCM. In DCM-related experimental models, NCOA4-mediated ferritin degradation can increase iron availability, whereas NCOA4 inhibition alleviates high-glucose-induced ferroptosis in cardiomyocytes Although irisin has been reported to inhibit NCOA4–FTH1-associated ferritinophagy and ferroptosis in a polycystic ovary syndrome model [39], whether exercise regulates this pathway in DCM remains unknown.

Exercise may also attenuate lipid peroxidation by improving antioxidant capacity and reducing oxidative stress. In high-fat diet-fed mice, aerobic exercise increased myocardial GPX4 activity and the GSH/GSSG ratio, reduced lipid peroxidation, and improved diastolic function [108]. High-intensity interval training (HIIT) attenuated myocardial fibrosis and iron deposition while increasing SLC7A11, GPX4, and SOD levels [105]. In a doxorubicin-induced cardiac injury model, eight weeks of aerobic exercise increased FTH1 and SLC7A11 expression and decreased ACSL4, Fe^2+^, ROS, and MDA levels [107]. Other studies have reported increased GPX4 and Nrf2 expression, reduced myocardial iron deposition and fibrosis, and improved cardiac function following exercise [95]. Human evidence has also indicated increased GPX4 expression and decreased hepcidin levels after exercise intervention [104]. Although these findings support the potential role of iron regulation and antioxidant defenses in exercise-mediated cardioprotection, their relevance to ferroptosis in DCM remains to be established.

Overall, aerobic exercise may alleviate ferroptosis-related myocardial injury through coordinated regulation of iron homeostasis and lipid redox balance. However, most available studies rely on total iron content, protein expression, and indirect markers of lipid peroxidation. Further studies should directly assess the LIP, mitochondrial iron pools, and NCOA4-mediated ferritinophagy, while using targeted interventions to determine whether these changes are necessary for the cardioprotective effects of exercise in DCM.

### 3.2. Exercise Suppresses Inflammation–Ferroptosis Crosstalk

Chronic low-grade inflammation contributes to DCM progression and may exacerbate oxidative stress and myocardial injury under conditions of metabolic dysfunction. Regular exercise can reduce systemic inflammation, improve insulin sensitivity, and attenuate metabolic disturbances, potentially limiting persistent inflammatory stimulation [110]. In DCM, hyperglycemia, lipid overload, and oxidative stress can activate the TLR4/NF-κB/NLRP3 signaling pathway, promoting the maturation and release of interleukin-1β (IL-1β) and IL-18 [111]. Palmitic acid may further disrupt inflammatory homeostasis by modulating TLR4-related signaling and activating JNK/p38 pathways under hyperglycemic conditions [112]. Consistent with these mechanisms, long-term exercise has been reported to suppress myocardial NLRP3 activation and attenuate inflammatory injury in DCM models [113]. Exercise has also been shown to reduce NLRP3-associated pyroptosis through the P2X4/PANX1–NLRP3 pathway, as indicated by reduced gasdermin D (GSDMD) cleavage and IL-1β maturation [114]. These findings support the anti-inflammatory effects of exercise in DCM, although they do not establish a direct effect on ferroptosis.

Importantly, ferroptosis and NLRP3/caspase-1/GSDMD-mediated pyroptosis are mechanistically distinct forms of regulated cell death. Ferroptosis is characterized primarily by iron-dependent lipid peroxidation and impaired antioxidant defense, whereas pyroptosis involves inflammasome activation, caspase-1-dependent GSDMD cleavage, membrane pore formation, and inflammatory cytokine release. Nevertheless, these pathways may interact through shared upstream stimuli, including oxidative stress, mitochondrial dysfunction, metabolic stress, and chronic inflammation. Thus, NLRP3 activation should not be considered a direct marker of ferroptosis; rather, sustained inflammasome activity and pyroptotic signaling may create an inflammatory and oxidative environment that increases susceptibility to ferroptosis. Exercise may consequently limit the interplay between these pathways by reducing inflammatory stress and improving redox homeostasis, although this interaction requires further direct validation in DCM. 

Exercise may therefore influence the potential interaction between inflammation and ferroptosis by reducing inflammatory stress and improving redox homeostasis. However, whether these effects are mechanistically linked in DCM remains unclear. Future studies should determine whether exercise-induced changes in inflammatory signaling contribute to the regulation of ferroptosis-related processes by simultaneously assessing inflammasome activation, lipid peroxidation, iron homeostasis, and key ferroptosis regulators. Targeted inhibition or activation of these pathways will also be needed to establish their causal relationships.

### 3.3. Exercise Improves Mitochondrial Homeostasis and Quality Control

Exercise is recognized as a potent physiological stimulus for mitochondrial biogenesis. Metabolic stress, mechanical stimuli, and transient changes in local oxygen availability during exercise can activate multiple intracellular signaling pathways, thereby promoting mitochondrial biogenesis and enhancing cellular energy metabolism [115,116]. In diabetic hearts, exercise training increases AMPK and PGC-1α signaling, mitochondrial DNA (mtDNA) content, and mitochondrial transcription factor A (TFAM) expression [22]. Accumulating evidence indicates that exercise activates the SIRT1/PGC-1α signaling pathway, thereby stimulating mitochondrial biogenesis and improving mitochondrial function [117]. In parallel, AMP-activated protein kinase (AMPK), a major cellular energy sensor and metabolic regulator, is rapidly activated by exercise and contributes to metabolic reprogramming and mitochondrial adaptation [118,119]. Similarly, exercise activates PGC-1α/Akt signaling, improves mtDNA replication and transcription, and preserves mitochondrial ultrastructure in DCM models [120]. Exercise has also been reported to improve mitochondrial oxidative phosphorylation capacity and membrane potential while reducing ROS production in diabetic hearts [121,122]. These findings provide evidence that exercise improves mitochondrial biogenesis and bioenergetic function in DCM. However, whether these adaptations directly mediate the suppression of ferroptosis requires further investigation.

Beyond mitochondrial biogenesis, exercise may maintain mitochondrial homeostasis by regulating mitochondrial dynamics and mitophagy. Exercise training modulates proteins involved in mitochondrial fusion and fission [123], while endurance exercise can activate PINK1/Parkin-dependent mitophagy and facilitate the removal of dysfunctional mitochondria [124]. In ischemic heart models, exercise-induced FNDC5/irisin–PINK1/Parkin signaling has been associated with enhanced mitophagy and improved cardiac function [125]. These findings support a role for mitochondrial quality control in exercise-induced cardioprotection, although evidence from ischemic models cannot be directly extrapolated to DCM. Moreover, mitophagy is context-dependent: inadequate mitochondrial clearance may promote the accumulation of damaged mitochondria, whereas excessive or dysregulated mitophagy may compromise mitochondrial integrity and cellular function [126]. A potential mechanistic link between mitochondrial quality control and ferroptosis involves Parkin-mediated regulation of acyl-CoA synthetase long-chain family member 4 (ACSL4). In an iron-overloaded cardiomyocyte model, Parkin promoted ACSL4 ubiquitination and reduced ferroptotic injury by modulating polyunsaturated fatty acid (PUFA)-phospholipid metabolism [78]. Because exercise can activate Parkin-dependent mitochondrial quality control, Parkin may represent a potential link between exercise-induced mitochondrial adaptation and the regulation of ferroptosis. However, whether exercise engages the Parkin–ACSL4 pathway in DCM has not been established. Thus, this mechanism should be considered a hypothesis supported by evidence from separate experimental models rather than a demonstrated pathway of exercise-mediated cardioprotection in DCM.

**Figure 3:**
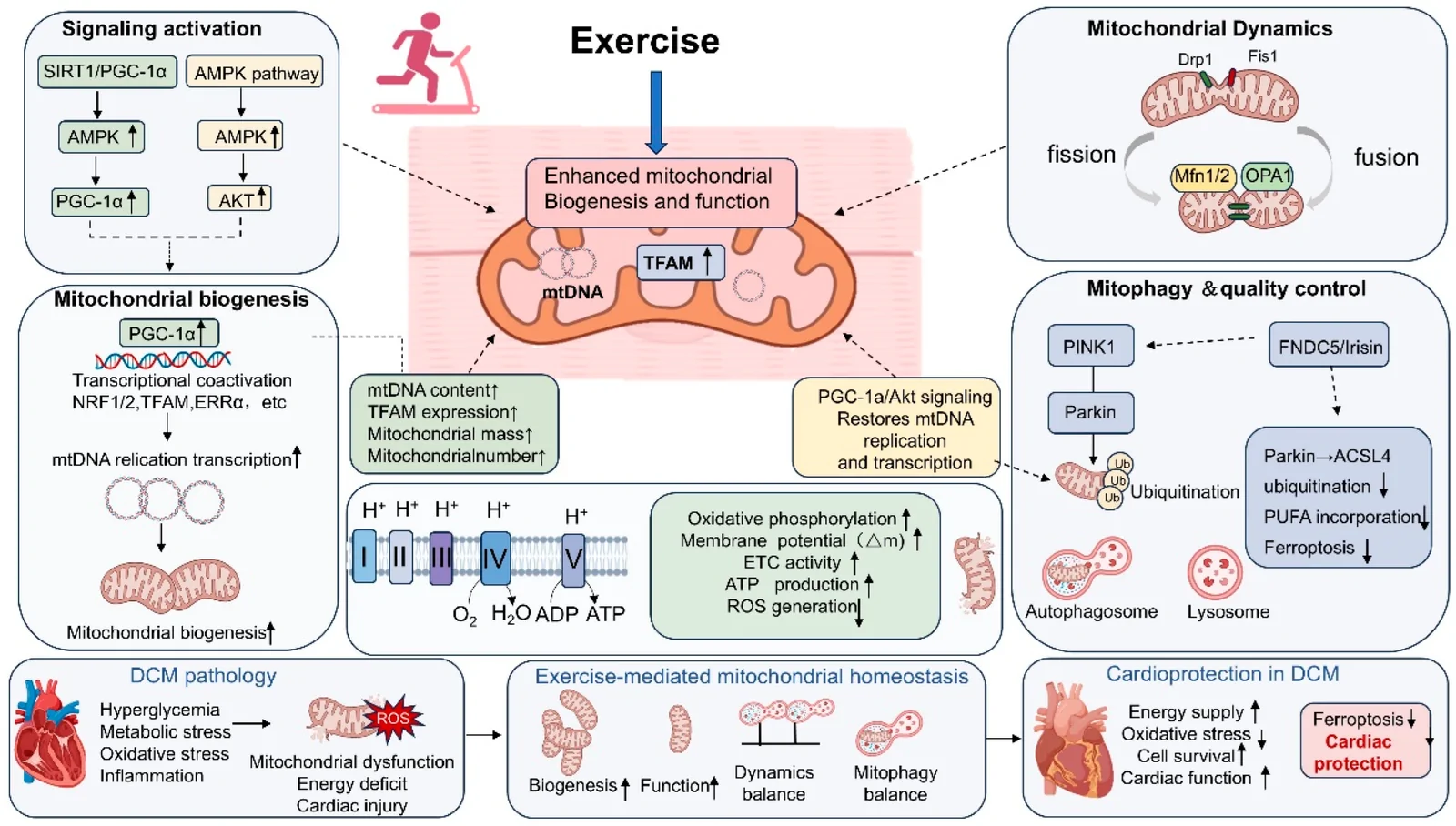
Exercise-Mediated Mitochondrial Homeostasis and Ferroptosis in Diabetic Cardiomyopathy Exercise promote mitochondrial biogenesis and function through SIRT1/PGC-1α and AMPK/AKT signaling, enhances mtDNA replication and mitochondrial mass, and improves oxidative phosphorylation and ATP production while limiting excessive ROS generation. In parallel, exercise maintains mitochondrial dynamics by balancing fission and fusion and enhances mitophagy and mitochondrial quality control through the PINK1/Parkin pathway. These coordinated adaptations restore mitochondrial homeostasis, alleviate oxidative stress and energy deficiency in DCM, and ultimately reduce ferroptosis susceptibility and promote cardiac protection. Vertical arrows denote the direction of change, with upward arrows (↑) indicating upregulation or enhancement and downward arrows (↓) indicating downregulation or inhibition. Abbreviation: ACSL4—acyl-CoA synthetase long-chain family member 4; AKT—protein kinase B; AMPK—AMP-activated protein kinase; ATP—adenosine triphosphate; DCM—diabetic cardiomyopathy; Drp1—dynamin-related protein 1; ERRα—estrogen-related receptor α; ETC—electron transport chain; Fis1—mitochondrial fission 1 protein; FNDC5—fibronectin type III domain-containing protein 5; LIP—labile iron pool; Mfn1/2—mitofusin 1/2; mtDNA—mitochondrial DNA; NRF1/2—nuclear respiratory factor 1/2; OPA1—optic atrophy 1; PGC-1α—peroxisome proliferator-activated receptor γ coactivator-1α; PINK1—PTEN-induced putative kinase 1; PUFA—polyunsaturated fatty acid; ROS—reactive oxygen species; SIRT1—sirtuin 1; TFAM—mitochondrial transcription factor A.

Collectively, exercise may improve mitochondrial homeostasis in DCM by coordinating mitochondrial biogenesis, oxidative phosphorylation, mitochondrial dynamics, and mitophagy. These adaptations may reduce excessive ROS production and improve cellular redox balance, thereby limiting conditions that favor lipid peroxidation. Although evidence supports the cardioprotective effects of exercise on mitochondrial function, the causal contribution of mitochondrial quality control and Parkin–ACSL4-mediated lipid remodeling to ferroptosis suppression in DCM remains to be determined (Figure 3).

### 3.4. Exerkine-Mediated Interorgan Communication and Regulation of Ferroptosis

The regulatory effects of exercise on ferroptosis are not restricted to intracellular signaling networks within cardiomyocytes. Increasing evidence suggests that interorgan communication mediated by exercise-responsive factors, collectively termed exerkines, represents an important mechanism underlying the systemic cardioprotective effects of exercise. Exerkines comprise a broad range of signaling molecules released from skeletal muscle, liver, adipose tissue, the heart, and other organs in response to acute or chronic exercise. These factors include myokines, hepatokines, adipokines, metabolites, and extracellular vesicles (EVs), which can act in autocrine, paracrine, or endocrine manners and circulate through the bloodstream to coordinate metabolic, inflammatory, and redox homeostasis across tissues [127]. These factors can act through autocrine, paracrine, or endocrine mechanisms to regulate systemic metabolism, inflammation, and redox homeostasis. Although these systemic adaptations may indirectly influence myocardial susceptibility to ferroptosis, direct evidence that exerkine-mediated interorgan communication suppresses ferroptosis in DCM remains limited. Therefore, this perspective provides a potential framework for understanding the systemic cardioprotective effects of exercise, but the specific factors, target organs, and molecular mechanisms involved require further investigation.

Fibroblast growth factor 21 (FGF21) is predominantly produced by metabolically active tissues, particularly the liver, and exerts its biological effects mainly through the FGFR1/β-Klotho (KLB) receptor complex. Emerging evidence indicates that exercise enhances myocardial responsiveness to FGF21 and activates the AMPK–FOXO3–SIRT3 axis, thereby improving mitochondrial function, reducing ROS generation, and alleviating lipid metabolic abnormalities in DCM [128]. Importantly, genetic studies have shown that either hepatic or systemic FGF21 deficiency, as well as cardiomyocyte-specific KLB deficiency, markedly attenuates the cardioprotective effects of exercise in DCM, suggesting that the liver-derived FGF21–myocardial KLB axis may represent an important endocrine pathway through which exercise mediates distal organ protection. Moreover, FGF21 has been reported to directly interact with ferritin and prolong its half-life, thereby suppressing ferroptosis in DCM. Activating transcription factor 4 (ATF4), an upstream regulator of FGF21 in DCM, further suggests the existence of an ATF4–FGF21–ferritin axis that contributes to cardioprotection through the regulation of ferroptosis and may represent a potential therapeutic target in DCM [129]. However, whether exercise suppresses ferroptosis and improves DCM specifically through exercise-induced elevation of FGF21 remains to be established.

Irisin is an exercise-responsive myokine encoded by fibronectin type III domain-containing protein 5 (FNDC5). It is a 112-amino-acid peptide generated by proteolytic cleavage of FNDC5 and can exert metabolic effects through binding to its receptor complex, including integrin αV/β5. Exercise promotes FNDC5 cleavage and the release of irisin from skeletal muscle, thereby increasing circulating irisin levels and facilitating communication with distant tissues involved in glucose and lipid metabolism, inflammation, and mitochondrial function. In a PA-induced in vitro model of type 2 diabetes-associated cardiomyopathy, irisin treatment markedly reduced ROS generation, intracellular Fe^2+^ and MDA levels, while increasing GSH levels and mitochondrial membrane potential (MMP), thereby suppressing ferroptosis and attenuating cardiomyocyte injury [106]. Consistently, exogenous irisin administration has been shown to inhibit ferroptosis and improve cardiac function in STZ-induced diabetic mice [130]. In DCM, swimming exercise increases irisin expression and activates the miR-34a-mediated SIRT1/PGC-1α/FNDC5 signaling pathway, thereby attenuating myocardial fibrosis [130]. Notably, exercise-induced increases in FNDC5/irisin are associated with improved myocardial mitochondrial homeostasis and cardiac function. Cao et al. [131] further demonstrated in hypoxic cardiomyocytes that FNDC5/irisin suppresses ferroptosis and improves mitochondrial dysfunction through the NRF2/HO-1 axis. Given that excessive mitochondrial ROS production, lipid peroxidation, and iron dyshomeostasis are major drivers of ferroptosis, the mitochondrial protective effects of irisin may indirectly reduce cardiomyocyte susceptibility to ferroptotic injury. Nevertheless, direct evidence demonstrating that exercise suppresses DCM-associated ferroptosis specifically through the FNDC5/irisin pathway remains limited.

Interorgan communication mediated by exercise-responsive factors may also involve extracellular vesicles (EVs) and their cargoes, particularly non-coding RNAs. EVs serve as important intercellular information carriers capable of transporting bioactive molecules, including miRNAs, proteins, and metabolites. Through circulation, EVs may transmit exercise-responsive molecular signals originating from skeletal muscle, liver, adipose tissue, and other organs to the myocardium, thereby contributing to cardiovascular metabolic adaptation and tissue remodeling. In DCM, several miRNAs have been implicated in myocardial fibrosis, angiogenesis, apoptosis, and ferroptosis. For example, miR-29b and miR-455 expression is markedly reduced in the myocardium of type 2 diabetic mice, whereas exercise training promotes the release of cardiovascular-derived EVs and increases the abundance of miR-29b and miR-455 within EVs, thereby attenuating myocardial fibrosis and suggesting that exercise may remodel the myocardial microenvironment through EV-mediated miRNA transfer [132,133]. Exercise interventions can also regulate other miRNAs associated with DCM progression. Ten weeks of swimming exercise combined with sodium butyrate treatment reduced myocardial miR-34a expression and promoted cardiac angiogenesis in type 2 diabetic mice [134]. In a type 1 diabetes model, both 5 weeks of high-intensity interval training and moderate-intensity continuous training reduced myocardial miR-1 expression and increased IGF-1 and IGF-1R levels, thereby attenuating cardiomyocyte apoptosis [135]. Exercise has also been reported to improve cardiac remodeling in type 1 diabetic cardiomyopathy through the miR-486a-5p/Mst1 signaling pathway [136]. Collectively, these findings indicate that exercise can remodel circulating and myocardial miRNA profiles and thereby influence multiple pathological processes involved in DCM.

More importantly, several miRNAs have been directly implicated in the regulation of myocardial ferroptosis. For instance, miR-671-5p has been reported to alleviate DCM-associated cellular injury by modulating ferroptosis-related molecules [137]. These observations raise the possibility that miRNAs may serve as molecular nodes linking exercise-induced interorgan communication to myocardial ferroptosis regulation. However, current evidence largely consists of two parallel lines of investigation: studies demonstrating that exercise modulates EV release and their miRNA cargoes, and studies showing that specific miRNAs regulate ferroptosis. Direct causal evidence establishing an integrated exercise–EV/miRNA–myocardial ferroptosis pathway remains limited. Therefore, exercise-derived EVs and their non-coding RNA cargoes may represent an important molecular bridge between interorgan communication and myocardial ferroptosis, providing a new perspective for understanding the cardioprotective effects of exercise in DCM and potentially informing the development of exercise-mimetic interventions and precision exercise therapies.

Other exercise-responsive factors, including IL-6 and adiponectin, may provide additional distal support for myocardial resistance to ferroptosis by modulating systemic metabolic and inflammatory states. Importantly, the acute increase in IL-6 induced by exercise differs biologically from the sustained elevation of IL-6 associated with chronic inflammation. During acute exercise, muscle-derived IL-6 contributes to fatty acid mobilization, hepatic glycogenolysis, and redistribution of energy substrates, whereas regular long-term exercise can reduce chronic low-grade inflammation, improve insulin sensitivity, and decrease ectopic lipid deposition. These adaptations may subsequently reduce myocardial lipotoxicity and oxidative stress, thereby indirectly limiting the metabolic conditions that favor ferroptosis [138]. Adiponectin, an important adipokine, promotes fatty acid oxidation and improves insulin sensitivity through activation of AMPK and related signaling pathways while exerting anti-inflammatory and antioxidant effects. Exercise-induced increases in adiponectin may therefore reduce myocardial lipid substrate overload and lipid peroxidation, thereby supporting myocardial redox homeostasis [139]. In addition, aerobic exercise has been reported to activate the PROC/PAR1/NRF2/HO-1 signaling axis, enhancing antioxidant defenses and alleviating myocardial injury in DCM [140]. Collectively, these findings suggest that exercise-responsive factors may not necessarily act directly on individual ferroptosis-associated molecules. Instead, they may reshape systemic energy metabolism, inflammatory signaling, and redox homeostasis, thereby reducing the metabolic and oxidative conditions that predispose the myocardium to ferroptotic injury. Such exerkine-mediated interorgan regulation may represent an important distal mechanism through which exercise protects the diabetic heart against ferroptosis.

## 4. Precision Exercise Strategies for Targeting Ferroptosis

### 4.1. Exercise Intensity and Ferroptosis Regulation

Exercise intensity influences physiological adaptation and cardioprotection. Its effects on ferroptosis may depend on the balance between exercise-induced oxidative stress and endogenous antioxidant defenses. Appropriate exercise can produce transient increases in ROS, which act as signaling molecules and activate adaptive pathways such as NRF2. Repeated exercise may therefore enhance antioxidant defenses and improve the ability of cardiomyocytes to remove lipid peroxides. In contrast, excessive exercise intensity or insufficient recovery may increase oxidative stress beyond the capacity of these defenses. This may promote lipid peroxidation and increase susceptibility to ferroptosis. Previous studies have shown that acute exercise can temporarily increase oxidative stress, whereas regular training can enhance antioxidant capacity through repeated exposure to controlled oxidative stimuli [141,142].

These responses are relevant to ferroptosis, which depends on the balance among iron availability, phospholipid peroxidation, and antioxidant defenses. Regular exercise may enhance NRF2-related signaling and the expression of antioxidant proteins, including SLC7A11 and GPX4. Exercise-induced mitochondrial adaptations may also improve oxidative metabolism and reduce excessive ROS production under resting conditions [143]. However, higher exercise intensity does not necessarily produce greater protection against ferroptosis. Although high-intensity exercise may induce stronger adaptive responses, its effects may depend on training status, metabolic health, and recovery. In DCM, pre-existing mitochondrial dysfunction and impaired antioxidant defenses may increase vulnerability to additional oxidative stress. Whether high-intensity exercise aggravates myocardial lipid peroxidation under these conditions remains to be established.

Evidence from general exercise physiology also indicates that high-intensity exercise does not invariably impair redox balance. A systematic review found that high-intensity exercise can induce substantial but generally transient oxidative stress, while trained individuals may exhibit greater antioxidant adaptive capacity [144]. Similarly, a meta-analysis by Torres-Rojo et al. reported that vigorous exercise could improve antioxidant capacity and reduce pro-oxidant markers in healthy adults [145]. These findings suggest that the effects of exercise intensity depend on several interacting factors rather than intensity alone. However, evidence from healthy adults cannot establish the optimal intensity for preventing ferroptosis in DCM.

From a clinical perspective, exercise intensity should be selected according to established recommendations and individualized assessment of cardiovascular status, functional capacity, comorbidities, and exercise tolerance. Moderate-intensity aerobic exercise may be an appropriate option for many individuals with diabetes, whereas HIIT may be considered for selected individuals who can tolerate it. These general exercise approaches should not be interpreted as ferroptosis-specific prescriptions. Whether different exercise intensities produce distinct effects on myocardial ferroptosis in DCM remains uncertain.

### 4.2. Exercise Duration and Cumulative Effects

Compared with exercise intensity, exercise duration and training duration may be more critical in determining whether the anti-ferroptotic effects of exercise can progress from transient stress responses to sustained myocardial adaptations. A single bout of exercise can induce transient oxidative stress and metabolic fluctuations, whereas repeated exercise over several weeks can promote cumulative adaptations that progressively improve iron homeostasis, antioxidant defenses, lipid metabolism, and mitochondrial function, thereby reducing myocardial susceptibility to ferroptosis. Thus, exercise duration should not be considered solely in terms of the duration of an individual exercise session but should also encompass the overall training period and cumulative exercise load. Emerging evidence from DCM models supports this concept. Recent studies have shown that 8 weeks of aerobic exercise attenuated myocardial oxidative stress and ferroptosis through the P4HA1/HIF-1α/NRF2/GPX4 axis, accompanied by improved cardiac function [26], suggesting that molecular adaptations induced by sustained training may represent an important basis for the anti-ferroptotic effects of exercise. In another study, 8 weeks of exercise training promoted the expression of FTH1, SLC7A11, and GPX4 through NAT10-mediated ac4C RNA modification, thereby suppressing myocardial ferroptosis in DCM. Notably, this study also identified reduced NAT10 and ac4C levels in cardiac tissues from individuals with DM and DCM, providing further evidence linking exercise-responsive molecular adaptations to the pathological context of human DCM [25].

Acute and chronic exercise may therefore exert their effects through distinct but interconnected adaptive processes. Acute exercise is characterized predominantly by transient signaling activation and stress responses, whereas prolonged training increasingly induces structural and functional adaptations. With progression of the training period, repeated exercise stimuli may sustain activation of antioxidant pathways such as NRF2 and enhance endogenous defense systems, while simultaneously improving mitochondrial oxidative phosphorylation efficiency and reducing excessive basal ROS production. In parallel, long-term exercise may improve insulin sensitivity and lipid metabolism and thereby limit myocardial fatty acid overload and reduce the availability of PUFA-containing lipid substrates susceptible to peroxidation. Accordingly, the anti-ferroptotic effects of exercise may gradually evolve from enhancement of antioxidant defense toward broader remodeling of iron metabolism, lipid metabolism, and mitochondrial homeostasis.

Importantly, however, extending the training period does not necessarily result in continuously increasing cardioprotective benefits. The myocardial protective effects of exercise are subject to a dose-dependent relationship. When cumulative training load chronically exceeds the individual’s capacity for recovery, or when exercise duration is mismatched to the individual’s adaptive capacity, persistent oxidative stress, inflammation, and mitochondrial injury may exceed endogenous compensatory mechanisms. This may impair exercise-induced antioxidant adaptation and increase the risk of lipid peroxidation, thereby potentially attenuating or counteracting the inhibitory effects of exercise on ferroptosis [146]. Therefore, exercise duration and intensity should be regarded as interdependent dimensions of the overall exercise dose, rather than as isolated variables.

Current studies investigating ferroptosis in DCM have predominantly adopted animal training protocols lasting approximately 4–12 weeks. However, direct comparisons among different training durations and longitudinal assessments at multiple time points remain scarce. Consequently, it remains unclear when ferroptosis-related molecular adaptations first emerge, when they reach a plateau, and whether prolonged training may eventually produce diminishing or even adverse effects. Future studies should therefore prioritize standardized reporting of exercise dose across experimental models and incorporate serial time-point assessments to delineate the temporal dynamics of exercise-induced adaptations in ferroptosis-related pathways. Such approaches may help define the optimal training window and cumulative exercise dose required to achieve sustained anti-ferroptotic cardioprotection in different stages of DCM.

### 4.3. Potential Differences Among Exercise Modalities

Different exercise modalities produce distinct physiological and metabolic stimuli. Aerobic exercise, RE, and HIIT may therefore affect ferroptosis-related pathways through different mechanisms. However, the available evidence varies substantially among modalities, and findings from non-cardiac models or other cardiac diseases should not be interpreted as direct evidence of anti-ferroptotic effects in DCM.

Aerobic exercise is the most extensively investigated modality in this field. In a high-fat diet-induced sarcopenia model, aerobic exercise activated AMPK/ACC signaling, improved iron regulation and antioxidant status, and reduced muscle loss and lipid accumulation [147]. In an aging-heart model, aerobic exercise was associated with PINK1/Parkin-mediated mitophagy, improved mitochondrial quality control, and the regulation of iron homeostasis [148]. These findings provide indirect mechanistic support but do not establish the same effects in DCM. More directly, aerobic exercise in DCM models enhanced NRF2/GPX4 signaling through the P4HA1/HIF-1α axis and improved cardiac structure and function [26]. In parallel, aerobic exercise may promote the expression of ferroptosis-related genes, including FTH1, SLC7A11, and GPX4, through NAT10-mediated ac4C RNA modification, thereby suppressing myocardial ferroptosis in DCM [25]. Collectively, these findings suggest that aerobic exercise may influence ferroptosis-related processes through antioxidant defenses, mitochondrial function, and iron regulation. Further studies are needed to determine the relative contributions of these mechanisms.

Direct evidence regarding RE and ferroptosis in DCM remains limited. Its potential benefits may arise partly from improvements in skeletal muscle function and systemic glucose and lipid metabolism. RE can increase muscle strength and glucose uptake and may improve insulin sensitivity, thereby reducing some of the metabolic disturbances associated with diabetes. These effects may indirectly influence myocardial lipid metabolism and oxidative stress. For example, altered ACSL4 activity and phospholipid composition can affect the availability of substrates for lipid peroxidation, but whether RE modifies these processes in DCM has not been established.

Zhong et al. [149] reported that resistance training increased glucose uptake, improved the lipid profile, and enhanced myocardial GPX activity in rats with DCM. Takino et al. [150] found a positive association between lower-limb muscle strength and exercise tolerance in patients with DCM, although this association does not establish a direct effect of RE on cardiac function or ferroptosis. Moreover, the metabolic effects of RE may vary with training protocols and individual characteristics. Improvements in systemic insulin sensitivity are not consistently observed across studies [151]. These findings support further investigation of RE as part of a broader strategy to improve metabolic health, but its direct effects on myocardial iron handling, lipid peroxidation, and the SLC7A11–GSH–GPX4 system remain insufficiently characterized.

HIIT provides repeated high-intensity stimuli interspersed with recovery periods. These stimuli may induce adaptations in mitochondrial function, antioxidant defenses, and inflammatory signaling. In a heart failure model, eight weeks of HIIT reduced myocardial iron deposition and increased SLC7A11, GPX4, and SOD levels, while attenuating endoplasmic reticulum stress [105]. However, these results are indirect evidence for DCM. Studies in diabetic rat hippocampus and experimental osteoarthritis have also reported changes in mitophagy or ferroptosis-related pathways following HIIT [152,153]. Such findings suggest that its effects may depend on tissue type and disease context, rather than establishing a general anti-ferroptotic effect.

In DCM models, HIIT has been associated with improvements in inflammatory signaling, myocardial fibrosis, autophagy, and cardiac function. Ma et al. [154] reported that HIIT attenuated cGAS–STING signaling and profibrotic responses in the DCM myocardium. Other studies found additional benefits when HIIT was combined with liraglutide [155] or reported improvements in cardiac function associated with autophagy activation [156]. These findings support the cardioprotective potential of HIIT, but they do not by themselves establish that ferroptosis suppression mediates these effects. Direct studies linking HIIT-induced changes in core ferroptosis pathways to improved cardiac outcomes in DCM remain limited.

Overall, aerobic exercise, RE, and HIIT may influence myocardial susceptibility to ferroptosis through partly different pathways. Aerobic exercise has the strongest direct evidence in DCM, whereas the potential effects of RE may involve systemic metabolic improvements and those of HIIT may involve combined metabolic, inflammatory, and mitochondrial adaptations. These differences provide a basis for comparative research but are not sufficient to establish the superiority of one modality or to support ferroptosis-based selection of exercise modalities in clinical practice.

### 4.4. Matching Exercise Prescription to Individual Metabolic Phenotypes

Patients with DCM may differ in the severity of insulin resistance, lipid dysregulation, oxidative stress, mitochondrial dysfunction, and iron abnormalities. These differences may influence their responses to exercise. It is therefore reasonable to investigate whether baseline metabolic characteristics help explain individual variation in exercise-induced cardioprotection. However, the use of ferroptosis-related phenotypes to guide exercise prescription remains an emerging research hypothesis rather than an established clinical approach.

From a mechanistic perspective, several hypotheses warrant investigation. In individuals with prominent iron dysregulation, future studies could examine whether exercise-induced changes in iron uptake, storage, and export are associated with myocardial outcomes. Relevant pathways include TFR1, FPN1, hepcidin, and NCOA4-mediated ferritinophagy. In individuals with pronounced lipid accumulation or lipotoxicity, studies could investigate whether changes in fatty acid metabolism, ACSL4-related phospholipid remodeling, and GPX4-dependent antioxidant defenses help explain differences in exercise responses. In those with mitochondrial dysfunction and elevated oxidative stress, research could examine whether changes in mitochondrial function and NRF2–SLC7A11–GSH–GPX4 signaling are associated with cardioprotection.

These proposed relationships should not be interpreted as evidence that particular phenotypes require specific exercise modalities or intensities. For example, combining aerobic and resistance exercise may improve glucose regulation, lipid metabolism, and physical function, but its effects on myocardial ferroptosis have not been established as a basis for individualized prescription. Similarly, moderate-intensity exercise may be appropriate for many individuals with limited exercise tolerance, while HIIT may be suitable for selected patients following clinical assessment. These decisions should currently be guided by established diabetes and cardiovascular exercise recommendations, disease stability, functional capacity, and individual risk, rather than by unvalidated ferroptosis-related biomarkers.

Future research should determine whether baseline iron status, lipid peroxidation, antioxidant capacity, mitochondrial function, or other biomarkers can predict differential responses to exercise. Prospective studies should compare exercise modalities and doses across well-characterized metabolic phenotypes and assess both ferroptosis-related outcomes and clinically meaningful cardiac endpoints. Where feasible, mechanistic interventions should be used to establish whether changes in ferroptosis pathways contribute causally to exercise-induced cardioprotection.

Ultimately, ferroptosis-informed precision exercise may provide a framework for investigating individual variation in exercise responses in DCM. However, its clinical value will depend on the validation of reliable biomarkers, reproducible phenotype-specific responses, and demonstrable benefits beyond conventional clinical assessment. Until such evidence becomes available, phenotype-guided exercise strategies should remain research hypotheses rather than evidence-based clinical algorithms.

## 5. Clinical Translation and Future Perspectives of Exercise-Mediated Ferroptosis Regulation

### 5.1. Ferroptosis-Related Biomarkers and Evaluation of Exercise Responses

The clinical translation of exercise-mediated ferroptosis regulation in DCM requires reliable methods to assess ferroptosis-related changes and monitor responses to exercise. However, no single biomarker can specifically identify ferroptosis in the human myocardium. Commonly measured variables, including ROS, MDA, 4-HNE, GPX4, and SLC7A11, also reflect other processes, such as oxidative stress, iron metabolism, and antioxidant defense. Therefore, changes in these markers should not be interpreted as definitive evidence of ferroptosis. A more reliable assessment should integrate evidence of iron dysregulation, lipid peroxidation, and impaired antioxidant defense, with additional functional or intervention-based evidence where available [157] (Table 2).

First, iron homeostasis-related biomarkers may help assess the upstream processes associated with ferroptosis. Serum ferritin, serum iron, transferrin saturation (TSAT), hepcidin, and soluble transferrin receptor 1 (sTFR1) can provide information on systemic iron storage and transport, whereas tissue-level assessment of TFR1, FPN1, FTH1, FTL, and NCOA4 may help determine whether exercise limits expansion of the LIP by regulating iron uptake, storage, export, and ferritinophagy. Importantly, total tissue iron content should be distinguished from the LIP. Total iron abundance does not necessarily reflect cellular susceptibility to ferroptosis, whereas the redox-active LIP is more directly involved in Fe^2+^-dependent oxidative reactions and may therefore represent a critical intermediate linking dysregulated iron metabolism to lipid peroxidation. Recent studies in DCM have reported ferroptosis-associated alterations characterized by increased Fe^2+^ levels, reduced FTH1 and SLC7A11 expression, and increased ACSL4 and PTGS2 expression [158]. These findings indicate that assessment of total tissue iron alone is insufficient to comprehensively characterize the ferroptotic state.

Second, lipid peroxidation and antioxidant defense markers may help characterize processes involved in ferroptosis. MDA and 4-HNE reflect the extent of lipid oxidative damage but lack sufficient specificity for ferroptosis and therefore should not be used as standalone indicators. In contrast, combined assessment of lipid-metabolism regulators, such as ACSL4 and the arachidonate lipoxygenase (ALOX) family, together with oxidized phospholipids and other lipid peroxidation products may provide a more informative evaluation of the effects of exercise on PUFA-containing membrane phospholipid remodeling and subsequent oxidation. Meanwhile, SLC7A11, the GSH/GSSG ratio, GPX4, and NRF2 provide complementary information regarding the cellular capacity to detoxify lipid peroxides and resist ferroptotic injury. Stronger evidence may be obtained when these findings are accompanied by functional experiments, such as rescue with ferroptosis inhibitors or targeted genetic interventions, where appropriate.

Ferroptosis-related markers should also be evaluated alongside cardiac structural, functional, and metabolic outcomes. For example, associations between changes in GPX4, SLC7A11, ACSL4, FTH1, NCOA4, and 4-HNE and changes in left ventricular ejection fraction (LVEF), diastolic function, myocardial fibrosis, insulin sensitivity, or exercise capacity may help determine whether ferroptosis-related changes accompany cardiac improvement. However, correlations do not establish that ferroptosis mediates the cardiac benefits of exercise. Circulating extracellular vesicles and oxidized lipids may also be explored as accessible candidates for monitoring exercise responses, but their ability to reflect myocardial ferroptosis remains to be established. Overall, current evidence is derived mainly from animal and cellular studies. Prospective human studies are needed to determine whether these markers can reliably reflect myocardial ferroptosis and predict cardiac responses to exercise.

### 5.2. Challenges, Clinical Translation, and Future Perspectives

Despite accumulating evidence that exercise can modulate ferroptosis-related pathways and protect the diabetic myocardium, several major challenges currently limit clinical translation. First, a substantial proportion of existing evidence derives from animal models or cultured cardiomyocytes, whereas direct evidence from patients with DCM remains limited. The pathophysiological complexity, comorbidities, medication use, age, sex, physical fitness, and baseline metabolic status of patients may substantially modify the response to exercise. Therefore, findings obtained from experimental models should not be directly extrapolated to clinical populations without appropriate validation. In particular, whether exercise-induced changes in ferroptosis-related pathways observed in experimental DCM are reproducible in patients and associated with clinically meaningful improvements in cardiac function remains to be established.

Second, the biological specificity of currently available ferroptosis biomarkers remains insufficient. Many commonly used indicators, including MDA, ROS, 4-HNE, ferritin, and GPX4, can be altered by oxidative stress, inflammation, mitochondrial dysfunction, or other forms of cell injury and are therefore not uniquely indicative of ferroptosis. Future studies should prioritize combinations of biomarkers that simultaneously capture iron accumulation, PUFA-phospholipid peroxidation, and impaired antioxidant defense, rather than relying on individual markers. The development and clinical validation of standardized and accessible biomarker panels will be important for determining whether ferroptosis-related alterations can be reliably detected and monitored in patients with DCM.

Third, the optimal exercise dose for modulating ferroptosis remains poorly defined. As discussed above, exercise intensity, duration, frequency, modality, and recovery interact to determine the overall biological stimulus. Importantly, the optimal dose may differ according to disease stage and individual metabolic phenotype. Future clinical studies should therefore move beyond conventional comparisons of “exercise versus control” and incorporate dose–response and phenotype-stratified designs. Integration of continuous physical activity monitoring, cardiopulmonary exercise testing, metabolic phenotyping, and ferroptosis-related biomarkers may help determine whether specific exercise characteristics are associated with more favorable molecular and cardiovascular responses.

Fourth, potential sex differences should be considered in future studies of exercise and ferroptosis in DCM. Sex-related differences in iron metabolism, lipid handling, oxidative stress, mitochondrial function, hormonal regulation, and susceptibility to cardiovascular injury may influence both ferroptosis-related responses and adaptation to exercise. However, whether and to what extent these factors modify exercise-induced regulation of ferroptosis in DCM remains insufficiently understood. Future clinical studies should therefore include adequate representation of both sexes and, where appropriate, perform sex-stratified analyses to determine whether ferroptosis-related biomarkers and cardiovascular responses to exercise differ between males and females.

Fifth, the interaction between exercise and pharmacological treatment requires further investigation. Patients with DCM frequently receive glucose-lowering, lipid-lowering, antihypertensive, and cardioprotective therapies, many of which can independently influence oxidative stress, mitochondrial function, inflammation, or iron metabolism. Exercise may therefore interact with these treatments in additive, synergistic, or potentially antagonistic ways. Understanding these interactions will be important for determining whether exercise can modify ferroptosis-related pathways in patients receiving standard medical therapy and whether such molecular changes translate into additional cardiovascular benefits.

Future research should increasingly adopt a multiscale and integrative approach. At the molecular level, studies should clarify how exercise simultaneously regulates iron trafficking, ferritinophagy, PUFA remodeling, lipid peroxidation, antioxidant defense, mitochondrial quality control, and inflammatory signaling. At the systemic level, the contribution of skeletal muscle, liver, adipose tissue, and circulating exercise-derived factors to myocardial ferroptosis should be investigated within an organ–organ communication framework. At the clinical level, longitudinal studies and randomized controlled trials are needed to determine whether changes in ferroptosis-related biomarkers are reproducibly associated with improvements in cardiac structure, function, and long-term cardiovascular outcomes.

Finally, an important unresolved question is whether exercise-induced modulation of ferroptosis represents merely a downstream consequence of improved metabolic and cardiovascular health or constitutes a causal mechanism underlying exercise-mediated cardioprotection. Establishing this distinction will require longitudinal biomarker studies, mechanistic intervention trials, and, where feasible, pharmacological or genetic modulation of ferroptosis pathways combined with controlled exercise interventions. Importantly, ferroptosis-based personalized exercise strategies should not yet be considered ready for clinical application. Their potential clinical utility will depend on further validation in human studies, including the establishment of reliable biomarkers, identification of clinically relevant ferroptosis phenotypes, characterization of sex-specific responses, and determination of appropriate exercise dose–response relationships. Ultimately, integrating ferroptosis biology with exercise physiology, metabolic phenotyping, multi-omics profiling, and clinical cardiology may provide a foundation for developing more individualized, mechanism-informed exercise strategies for DCM, but substantial clinical evidence is still required before such approaches can be translated into routine clinical practice.

## 6. Conclusions

Exercise may improve cardiac function in DCM and is associated with changes in iron homeostasis, lipid peroxidation, antioxidant defense, and inflammation. Experimental evidence suggests that ferroptosis-related processes may contribute to DCM progression and that exercise may modulate these processes through pathways involving NRF2, SLC7A11–GSH–GPX4, and ACSL4. However, the causal role of ferroptosis in the cardioprotective effects of exercise remains unclear. Most available evidence comes from animal and cellular studies, and the clinical validity of ferroptosis-related biomarkers has not been established. Future studies should combine comprehensive assessment of ferroptosis with targeted mechanistic interventions and well-designed human studies to clarify causal relationships and determine whether ferroptosis-related findings can inform individualized exercise strategies for DCM.

## Figures and Tables

**Table 1:** Evidence for Exercise-Mediated Regulation of Ferroptosis-Related Markers and Cardiac Outcomes Across Experimental Models and Human Studies.

Model/Population	Exercise Modality	Ferroptosis-Related Endpoints	Cardiac Outcomes	Evidence
DCM model [26]	Moderate-intensity aerobic treadmill exercise for 8 weeks	Increased GPX4, FTH1, FSP1; decreased ACSL4, and Fe^2+^	Increased EF and FS, Reduced LVIDd and LVIDs	Direct
DCM model [25]	Treadmill training for 8 weeks	Increased SLC7A11, GPX4 and FTH1; decreased PTGS2	Increased FS and E/e’	Direct
Myocardial I/R model [103]	Treadmill training for 6 weeks	Increased GPX4, SLC7A11, decreased ACSL4,	Increased EF and FS, Reduced LVIDd and LVIDs; reduced collagen deposition	Indirect
DCM model [95]	Moderate-intensity treadmill training for 12 weeks	Increased GPX4, Nrf2; decreased iron deposition	Increased EF and FS; attenuated cardiac fibrosis	Direct
Men recovering from addiction and receiving methadone maintenance therapy [104]	20-watt Ergoline bicycle ergometer, 5 min every two weeks, 20 min during weeks 1–2, 25 min during weeks 3–4, 30 min during weeks 5–6, and 35 min in the final two weeks (weeks 7–8).	Increased GPX4; decreased SLC7A11, hepcidin levels	NO	Emerging
HF model [105]	HIIT for 8 weeks	Increased SLC7A11, GPX4 and SOD; reduced iron deposition	Increased EF and FS; reduced cardiac fibrosis,	Indirect
(PA)-induced cardiomyocytes [106]	NO	Decreased ROS production, Fe^2+^ content, and MDA levels, Increased GSH levels and MMP, Xc-/GSH/GPX4	Alleviated myocardial injury	Emerging
Doxorubicin-induced cardiotoxicity (DIC) model [107]	Aerobic exercise for 8 weeks	Increased FTH1,SLC7A11; Decreased ACSL4, Fe^2+^, ROS, MDA	Increased LVEF and LVFS; decreased myocardial fibrosis and cTnT levels	Indirect

**Table 2:** Exercise-responsive ferroptosis-related biomarkers and their potential value for precision evaluation of exercise efficacy in diabetic cardiomyopathy.

Biomarker	Ferroptosis Relevance	Exercise-Related Evidence in DCM	Evidence Level
GPX4	Core Defense Against Lipid Peroxidation	Upregulates GPX4	Direct
SLC7A11	Regulates Cystine Uptake and GSH Availability	Enhances Antioxidant Defense	Direct/Mechanistic
ACSL4	Determines the Formation of Peroxidation-Susceptible PUFA-Containing Phospholipids	Downregulates ACSL4	Direct
NCOA4	Mediates Ferritinophagy	Limited Evidence for Exercise-Mediated Regulation	Emerging
TFR1/FPN1	Regulates Iron Uptake and Export	Improves Iron Homeostasis	Mechanistic
LIP/Fe^2+^	Directly Reflects the Labile Iron Pool	Limited Evidence in Exercise-Treated DCM	Emerging
4-HNE/MDA	Lipid Peroxidation Products	Reduces Their Levels	Indirect
Ox-PL	Ferroptosis-Associated Membrane Lipid Oxidation	Insufficient Evidence for Exercise-Mediated Regulation	Emerging
NRF2	Upstream Transcriptional Regulation of Antioxidant Defense	Activates NRF2	Mechanistic
PTGS2/CHAC1	Ferroptosis-Related Responses	Limited Evidence for Exercise-Mediated Regulation	Indirect

## Data Availability

All data from this study are included in this article.

## Abbreviations

AA—arachidonic acid; ABHD11—α/β-hydrolase domain-containing protein 11; ACSL4—acyl-CoA synthetase long-chain family member 4; AdA—adrenic acid; AGEs—advanced glycation end products; ALOX—arachidonate lipoxygenase; AMPK—AMP-activated protein kinase; AREs—antioxidant response elements; ATF4—activating transcription factor 4; BCAAs—branched-chain amino acids; DAMPs—damage-associated molecular patterns; DCM—diabetic cardiomyopathy; DM—diabetes mellitus; DMT1—divalent metal transporter 1; DOX—doxorubicin; Drp1—dynamin-related protein 1; ECM—extracellular matrix; ER—endoplasmic reticulum; EVs—extracellular vesicles; FGF21—fibroblast growth factor 21; Fis1—fission 1 protein; FNDC5—fibronectin type III domain-containing protein 5; FPN1—ferroportin 1; FTH1—ferritin heavy chain 1; FTL—ferritin light chain; GCLC—glutamate-cysteine ligase catalytic subunit; GCLM—glutamate-cysteine ligase modifier subunit; GPX4—glutathione peroxidase 4; GSDMD—gasdermin D; GSH—glutathione; HIIT—high-intensity interval training; HO-1—heme oxygenase-1; IL-1β—interleukin-1β; IL-6—interleukin-6; IL-10—interleukin-10; IL-18—interleukin-18; Keap1—Kelch-like ECH-associated protein 1; LIP—labile iron pool; LPCAT3—lysophosphatidylcholine acyltransferase 3; L-OH—lipid alcohols; L-OOH—phospholipid hydroperoxides; LOOH—lipid hydroperoxides; LOH—lipid alcohols; LVEF—left ventricular ejection fraction; MDA—malondialdehyde; MFN2—mitofusin 2; Mfn1/2—mitofusin 1/2; miRNAs—microRNAs; MMP—mitochondrial membrane potential; MRP1—multidrug resistance-associated protein 1; mtDNA—mitochondrial DNA; NAT10—N-acetyltransferase 10; NCOA4—nuclear receptor coactivator 4; NF-κB—nuclear factor-κB; NLRP3—NOD-like receptor family pyrin domain-containing 3; NRF2—nuclear factor erythroid 2-related factor 2; OPA1—optic atrophy 1; OXPHOS—oxidative phosphorylation; P2X4—purinergic receptor P2X 4; PA—palmitic acid; PANX1—pannexin 1; PGC-1α—peroxisome proliferator-activated receptor γ coactivator-1α; PE—phosphatidylethanolamine; PINK1—PTEN-induced putative kinase 1; PTGS2—prostaglandin-endoperoxide synthase 2; PUFA—polyunsaturated fatty acid; PUFAs—polyunsaturated fatty acids; RAGE—receptor for advanced glycation end products; RE—resistance exercise; ROS—reactive oxygen species; SIRT1—sirtuin 1; SLC3A2—solute carrier family 3 member 2; SLC7A11—solute carrier family 7 member 11; SOD—superoxide dismutase; sTFR1—soluble transferrin receptor 1; T2DM—type 2 diabetes mellitus; TF—transferrin; TFAM—transcription factor A, mitochondrial; TFR1—transferrin receptor 1; TGF-β—transforming growth factor-β; TLR4—Toll-like receptor 4; TNF-α—tumor necrosis factor-α; TSAT—transferrin saturation; UPR—unfolded protein response.
